# Repurposing the Knutsford-1 borehole as a deep borehole heat exchanger with consideration of palaeoclimate corrections to heat flow in the Cheshire Basin

**DOI:** 10.1038/s41598-025-29816-3

**Published:** 2025-11-29

**Authors:** Christopher S. Brown, Sean M. Watson, Isa Kolo, Luke Morrison, Andrew M. W. Newton, Gioia Falcone

**Affiliations:** 1 James Watt School of Engineering, University of Glasgow, Glasgow, G12 8QQ UK; 2 TownRock Energy, East Woodlands House, Dyce, Aberdeen, AB21 0HD UK; 3 School of Natural and Built Environment, Queen’s University Belfast, University Road, Belfast, BT7 1NN UK

**Keywords:** Knutsford-1 borehole, Borehole heat exchanger, Groundwater flow, Palaeoclimate corrections, Energy science and technology, Engineering, Energy infrastructure

## Abstract

Subsurface thermal data from UK boreholes typically lack palaeoclimatic corrections, leading to underestimations in heat flow. This can significantly affect predicted geothermal resources and system performance, creating a false perception of energy limitations. This study evaluates the impact of palaeoclimate corrections on geothermal performance in an onshore setting; focusing on the potential for a well to be re-entered and repurposed as a deep borehole heat exchanger (DBHE). Using the Knutsford-1 borehole, this re-evaluation for palaeoclimatic impacts on heat flow shows that corrected heat flows (52 mW/m^2^) exceed uncorrected values (46 mW/m^2^). In a steady state conductive model, temperature predictions based on the corrected heat flow align more closely to the recorded temperature data. Moreover, transient DBHE simulations using OpenGeoSys software over 25 years reveal a minimum 17 kW increase in thermal yield, highlighting the operational implications and benefits of these corrections. With thousands of legacy boreholes worldwide, integrating palaeoclimate corrections into geothermal assessments could reveal substantial untapped energy potential. By unlocking previously overlooked geothermal potential, this research highlights how accurate subsurface (re)assessments can transform legacy infrastructure into a cost-effective, sustainable energy source – demonstrating that with better data and a more holistic approach, existing wells can support low-carbon heat production.

## Introduction

Greenhouse gases trap heat radiated by the Earth within the atmosphere that would otherwise be emitted into space; without the greenhouse effect the average surface temperature of the Earth would be 33 °C lower than at present^1^. However, the burning of fossil fuels has greatly increased the presence of greenhouse gases (i.e., CO_2_) in the atmosphere, contributing to at least a 1 °C rise in the global average surface temperature since 1880^2^. In order to meet net zero targets by 2050, decarbonisation is, therefore, an essential part of developing a low-carbon energy portfolio^3^.

With current gas usage meeting 73% of the UK’s heat demand, alternative, renewable energy sources are required to lower the associated emissions. Thus, the heating sector represents a significant opportunity for decarbonisation efforts^4^, with geothermal energy providing a leading option due to its ability to provide a constant base load that is weather independent, unlike wind and solar, with minimal associated emissions^5^. At present, deep geothermal development in the UK is in its infancy. There is only: (i) one commercial deep (> 500 m) open-loop geothermal system operating in Southampton (Wessex Basin); and (ii) one commercial deep closed-loop system at the Eden Project in Cornwall^6^. While further open-and closed-loop deep systems are currently under development (such as the United Downs Project), or at pilot stage (e.g., Kirby Misperton), there remains significant potential for redeveloping legacy subsurface infrastructure, such as ex-oil and gas wells (e.g.^7,8^).

**Fig. 1:**
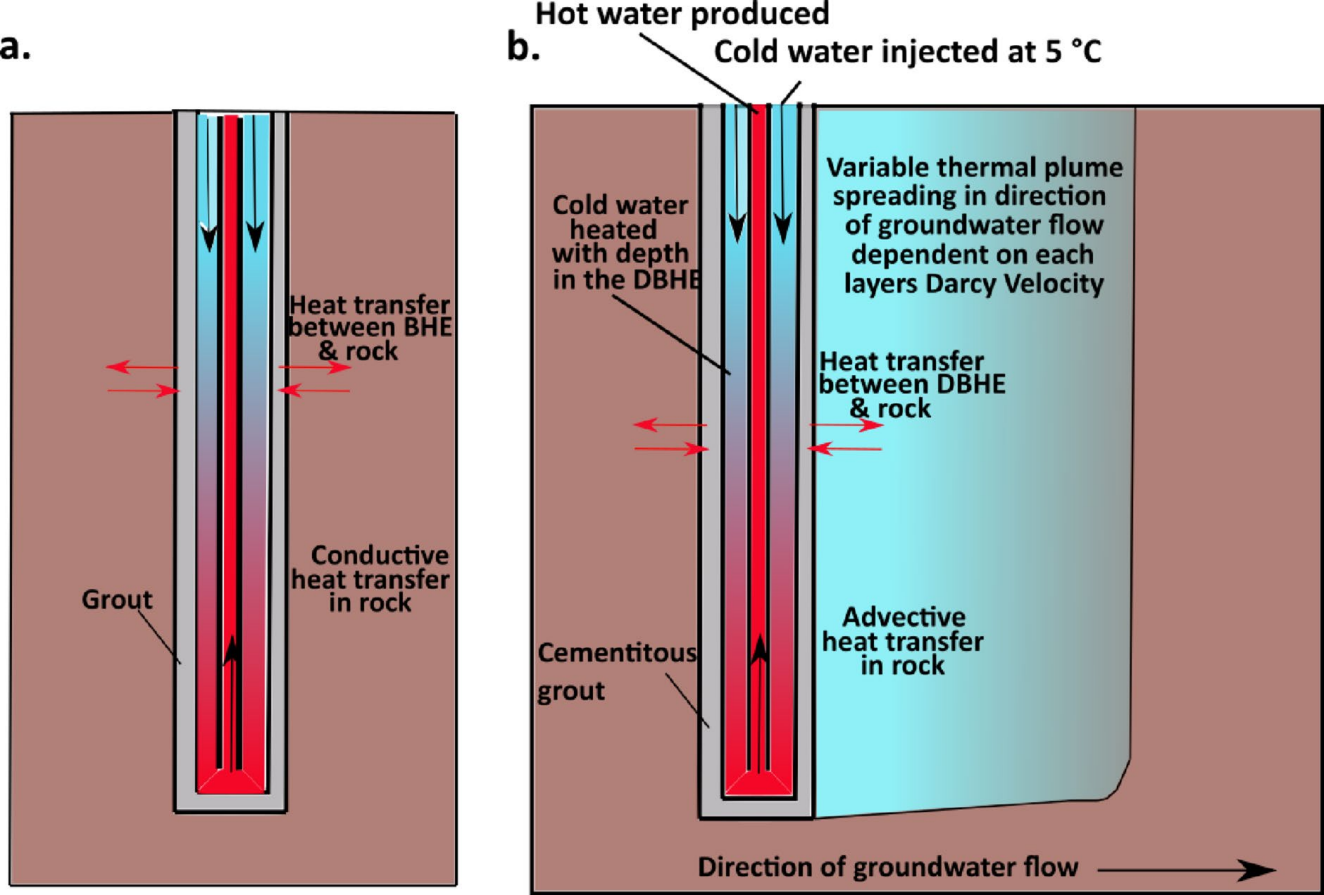
Schematic of deep borehole heat exchanger (DBHE) operating with (**a**) conductive and (**b**) advective (forced through groundwater flow) heat transfer in the subsurface.

This paper focuses on deep borehole heat exchangers (DBHEs); which are closed-loop systems that involve the circulation of fluid in a closed circuit whereby the fluid is continuously reused. Cold heat transfer fluid is circulated down the annular space, warming with depth under the natural geothermal gradient via conduction through the borehole wall, before being circulated to the surface through the central pipe (Fig. 1a). DBHEs have poorer heat recovery compared to open-loop geothermal systems, as the dominant mechanism of heat transfer is conduction in closed-loop rather than advection in open-loop^9^; however, they can help to minimise geological risk as there is no hydraulic interaction between the DBHE and reservoir, no reliance on permeability, and low parasitic loads from associated circulation and heat pumps^10^. Given the UK’s industrial legacy of onshore hydrocarbon exploration, there has been significant interest in the repurposing of hydrocarbon wells^7^, shale gas wells^11^ and, more recently, disused exploratory geothermal wells^12–15^.

Recent advances focusing on repurposing and new installations of DBHEs have been summarised in several review papers^16–18^. New projects have been developed, which have required support from modelling studies due to the paucity of data available. Numerical and analytical modelling studies have investigated the influence of geological and engineering parameters on system performance (e.g^19–24^), optimisation (e.g^25–27^), and integration with power generation, or heating and cooling systems at the surface level (e.g^28–30^). However, as far as the authors are aware, there has yet to be a study which considers factors which perturb steady-state (initial) conditions in the subsurface, and thus the influence on steady-state and transient modelling of DBHE systems. This study focuses on one particular factor, and that is the need to account for the effect of past climatic changes on heat flow, and the subsequent effect this has on modelling the performance of DBHE systems which will be key to consider for carbon mitigation strategies. Heat flow refers to the ability to transmit heat through the crust as a function of the geothermal gradient and thermal conductivity. Past studies of DBHEs have neglected the influence of past climate changes and typically, either model a linear constant geothermal gradient, or a steady-state set up which does not account for such factors and use the regional basal heat flow for the area. This can lead to underestimation of energy within the system available for heat extraction, which could be pivotal in determining the amount of heat that can thus be extracted.

The Earth has undergone a number of climatic changes over millions of years, the most notable in this context is the icehouse conditions of the Pleistocene epoch (2.58 million to 11,700 years ago). During this period, there were multiple glacial-interglacials cycles, and while there is uncertainty on the magnitude of the glacial stages within each cycle, there is clear evidence that the British Isles regularly experienced ice-covered conditions^31,32^. It is known that past climate change has affected the temperature at the Earth’s surface and thus the temperature distribution at depth. This, in turn, has affected the geothermal gradient and heat flow within the Earth’s crust. Thus, in areas such as the British Isles, where conditions during Pleistocene cold stages were particularly severe, there is a need to consider this palaeoclimatic effect on heat flow and the geothermal gradient when characterising geothermal resources for contemporary exploitation^33^. The effect that changes in the climate over past millennia have on heat flow data has been known for some time and was first factored into data by Benfield^34^, who calculated the effect that the last period of ice cover had on heat flow data across various UK boreholes. Despite this acknowledgment, palaeoclimate corrections have not been factored into heat flow analyses, and subsequent transient geothermal system analyses, in many studies since. Beck^35^ attributed this to difficulties in determining temperature evolution through multiple glacial-interglacial cycles of variable ice sheet duration and magnitude. The significance of this palaeoclimatic effect on heat flow is profound as Westaway and Younger^33^, and Watson^36^, estimate palaeoclimate corrections in the UK up to 20 mW/m^2^, meaning that the UK’s heat flow and geothermal base are likely to be underestimated. Westaway and Younger^33^ also point out numerous examples of studies which have decided to neglect palaeoclimate corrections or account for them in a somewhat unsatisfactory way.

The chronic failings in the past to properly correct for the palaeoclimate effect has resulted in systematic errors in heat flow data and thus the UK’s geothermal potential has been downplayed, and only recently has this begun to be rectified. Palaeoclimate corrections have seldom been applied to geothermal problems, let alone the impact on closed-loop deep borehole heat exchangers where the operational capacity of systems is low in comparison to open-loop systems. Therefore, the impact of incorrect heat flow estimates could drastically reduce the anticipated thermal power output, and thus adversely impact the perceived feasibility of developing such systems. This is of particular importance to areas of the country where heat flow data is scarce, or confidence in accuracy of data is low (i.e., in the Cheshire Basin).

This paper targets the potential to repurpose a hydrocarbon exploration borehole as a DBHE. The borehole in question is named Knutsford-1, and was drilled in the Cheshire Basin, through a thick Permo-Triassic sedimentary sequence, of which numerous aquifers are present which could yield significant groundwater flows^37^. The Cheshire Basin is one of several deep sedimentary basins in the UK which host deeply buried aquifers which are prospective targets for geothermal exploration, although their deep potential is largely untested^38^. The basin contains up to 4.5 km of Permian and post-Permian sedimentary rocks, which in turn are underlain by hundreds of metres of Carboniferous strata^39^. Over much of the basin, the geothermal gradient lies between 20 and 22 °C/km, and estimates of heat flow range from 25 to 59 mW/m^2^^34,40,41^. These disappointing geothermal gradient and heat flow estimates from boreholes within the Cheshire Basin may be explained by the aforementioned residual depression of geothermal gradient, and therefore heat flow estimates, in the uppermost Earth’s crust due to severe cooling experienced during periods of Pleistocene glaciation^33,42^.

The neglection, or under-appreciation, of this palaeoclimatic effect on heat flow is the key focus of this paper, which will address three key aims: (1) to understand how past glaciations impact heat flow and how this may impact the steady state and transient modelling of the performance DBHEs, (2) investigate the influence of groundwater flow in multiple aquifers on the DBHE performance (Fig. 1b), and, (3) establish optimal engineering parameters for the Knutsford-1 borehole to assess its potential for repurposing as a DBHE. These aims will be investigated through a detailed modelling study that incorporates palaeoclimate correction methods from Westaway and Younger^33^, and Watson^36^, and uses OpenGeoSys (OGS) to simulate transient heat flux in the subsurface for the DBHE and surrounding rocks (e.g^12,13,21,43,44^).

## Geological overview of the Cheshire basin

The Cheshire Basin is a target for geothermal exploration in the UK due to: (i) the high-quality hydraulic characteristics of the aquifers, (ii) the substantial thickness of aquifers (up to 2 km), (iii) the significant geothermal resource associated to deep sandstones up to 4.5 km^38,45–47^ and (iv) moderate temperature gradients up to 27 °C/km in some areas of the basin^48^. Further localised transient numerical modelling of thermal and hydraulic processes under operating conditions also highlights the potential to develop the basin for open- and closed-loop systems^8,22,49,50^, although at depth the hydraulic potential for open-loop development remains untested. The basin formed following rifting of Pangaea^51,52^ and is an asymmetrical half graben with sedimentary infill predominantly composed of Permo-Triassic fluvial to aeolian sandstones, overlain by mudstones deposited in the Late Triassic and Jurassic during a marine transgression^53–56^. Recent deposits are characterised by Quaternary glacial sediments that were either delivered or reworked by ice during the last glacial period^39^.

Localised basin studies from Plant et al.^39^ form the best hydrogeological assessment of the basin. Most recharge reaches the upper most Sherwood Sandstone Group through thick covers of low permeability drift, with the main sources of recharge from rainfall, with some contribution from leaking sewers, water mains and from the River Mersey. They also highlight that there are very slow residence times for deeper saline aquifers (> 500 m depth) providing evidence for generally slow-moving groundwater flows which are captured within the numerical model.

**Fig. 2:**
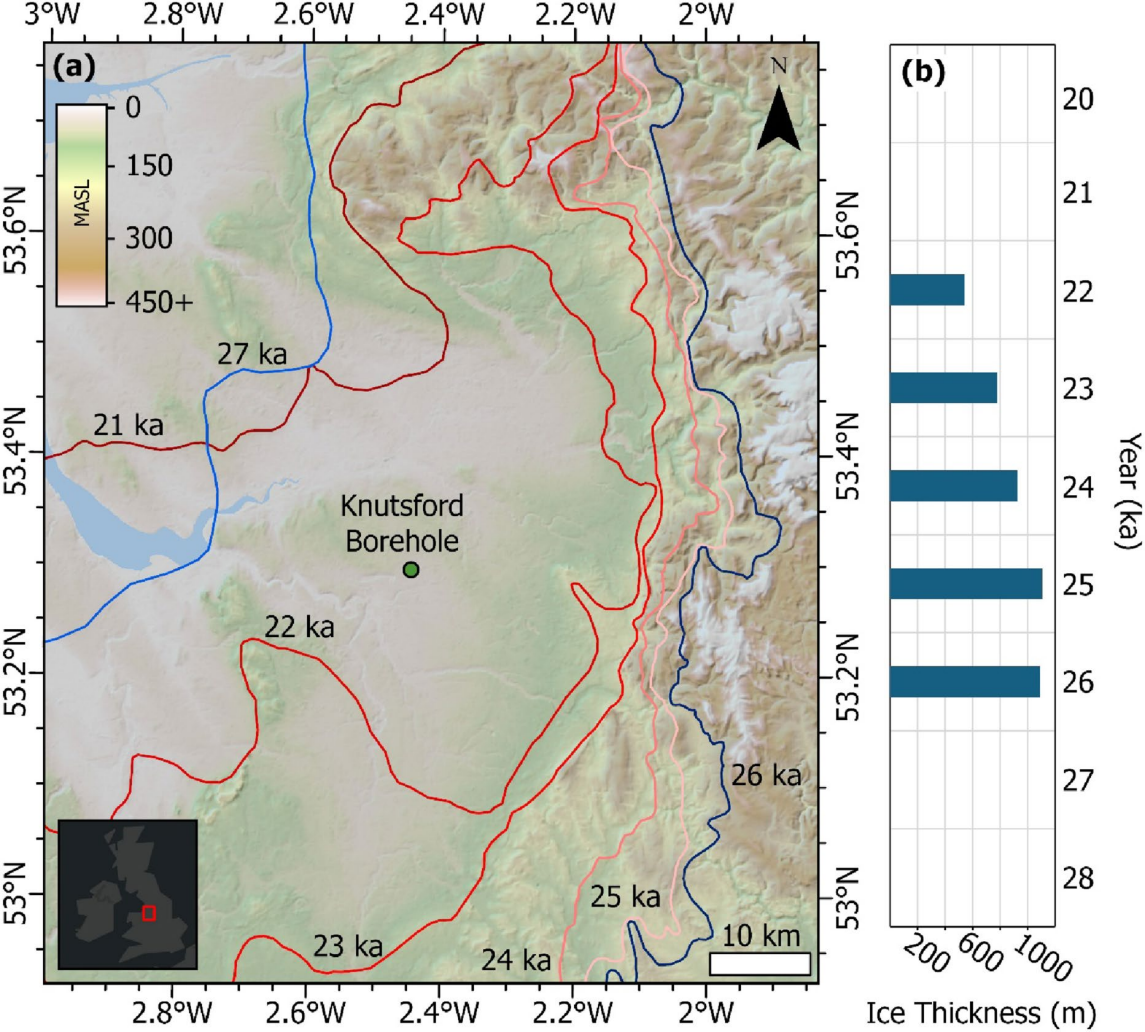
(**a**) map showing the ice margin evolution through the last glacial cycle in relation to the location of the borehole studied here. Blue margin positions characterise the advance of the ice sheet from 27 ka to 26 ka, while red margins capture the retreat from the maximum position, 25 ka to 21 ka. The small inset in the lower left shows the location of the map at the national scale. On the key, MASL means Metres Above Sea Level and capture the elevation of the topography. (**b**) Graph showing modelled ice thickness in metres over the borehole study site for different timestamps of the last glacial cycle. These thicknesses estimates have been derived from the outputs of Clark et al.^59^.

While there is sedimentological evidence that the Cheshire Basin has been glaciated prior to the last glacial cycle (e.g^57^), recent modelling of the evolution of the British-Irish Ice sheet through the last glacial, constrained by extensive fieldwork and geochronological constraints, allow for an in-depth insight into the evolution of the ice sheet margin (Fig. 2). The modelling outputs also allow for an estimate on the possible thickness of the ice at the Knutsford-1 borehole study site through this same period (Fig. 2). This work shows that through the last glaciation, glaciofluvial deposits observed beneath a subglacial till suggest that ice advanced from the northwest of the study area into the Cheshire Basin by ~ 27 ka and continued to advance as an ice lobe that extended to north of Birmingham by ~ 26 ka^58–60^. Ice margins reached their maximum limit in the study area by ~ 25 − 24 ka before a broadly monotonic style of retreat to the northwest is interpreted, with the ice margin reaching the contemporary coastline by ~ 21 ka^58,59,61^. By 20 ka the main ice margin had retreated from the Cheshire Basin, with the main ice margin separating from the Welsh Ice Cap and being located to the north and northwest in the Lake District and Irish Sea, respectively^59^. Temperature reconstructions for the latter part of the deglaciation show stadial-interstadial mean July temperature swings of up to 10 °C, with maximum temperatures of up to ~ 20 °C prior to cooling, before the onset of the Holocene is marked by an abrupt temperature increase of 9 °C^62^. The early part of the Holocene reflects the post-glacial warming, before the Holocene Climatic Optimum in the mid-Holocene, with the latest Holocene characterised by centennial-scale climatic oscillations, such as the Bronze Age Warm Period and the Little Ice Age^63^.

## Methods

This study integrates palaeoclimate corrections into steady-state and transient numerical models to determine the impact of using uncorrected and corrected heat flow values for modelling of DBHEs. Detailed methods are thus presented herein, with palaeoclimate corrections adopting the methods from Westaway and Younger^33^, and Watson^36^, and the transient numerical modelling using OGS.

### Palaeoclimate corrections

#### Uncorrected heat flow methodology

The aim of this study is to apply a palaeoclimate correction to heat flow derived from the Knutsford-1 borehole. As a precursor to this, we first calculate the uncorrected heat flow for the Knutsford-1 borehole, which has hitherto not been determined. To do this, we use the Bullard method^64^, presented in Eq. 1:

1 $$\:{T}_{z}\:=\:{T}_{0}+Q\:\sum\:_{i=1}^{n}\frac{\varDelta\:{z}_{i}}{{\:\lambda\:}_{i}}$$

The Bullard method is based upon the concept of thermal resistance (°C/W) (i.e., the reciprocal of the thermal conductivity, $\:\lambda\:$, (W/m °C) integrated over depth *z* (m)). Heat flow, *Q*, (mW/m^2^) is estimated by a linear relationship between the thermal resistance and the measured subsurface temperature, *T*, (°C).

Application of Eq. 1 involves calculating the thermal resistance to each depth where a temperature measurement has been observed. This requires the subsurface temperature measurements to be known, alongside the lithological log of the borehole in question, and either measured or assumed values of thermal conductivity for each lithology encountered in the borehole.

Subsurface temperature measurements were observed at various depths from 715 to 3039 m within the Knutsford-1 borehole. In addition, we calculate a representative ground surface temperature to enable determination of uncorrected heat flow across the full extent of the stratigraphic sequence encountered by the borehole (e.g., from ground surface to total depth). The ground surface temperature was calculated as 9.8 °C. This is the mean annual surface air temperature for 1974 of 9.6 °C observed at the Ringway meteorological station^65^, with a lapse rate of 8 °C/km^66^ applied to account for the difference in elevation between the Ringway meteorological station and the elevation of the ground surface at the location of the Knutsford-1 borehole.

As thermal conductivity was not measured, either in-situ, or from core or cuttings, we assign representative values of thermal conductivity to each lithology encountered between 0 and 3039 m described in the Knutsford-1 borehole lithological log.

The values of thermal conductivity and temperature used to derive the uncorrected heat flow for the Knutsford-1 borehole are shown in Tables 1 and 2, respectively. Based upon the assumed values of thermal conductivity, and the respective thicknesses of the lithologies encountered in the Knutsford-1 borehole, the cumulative thermal resistance has been determined throughout the borehole coincident with the depths of temperature measurement (Table 2).

**Table 1:** Thermal properties assigned to lithologies encountered in the Knutsford-1 borehole.

Property	Quaternary	Claystone	Coal	Limestone	Mudstone	Salt	Siltstone	Sandstonest
$\:\lambda\:$ (W/m °C)	0.77^e^	2.12^d^	0.31^d^	3.32^d^	1.88 ^d^	4.87 ^d^	2.22 ^d^	3.41 ^d^
c (J/kg °C)	860^a^	860 ^a^	1300 ^a^	880 ^a^	770^b^	880 ^a^	910 ^a^	775 ^a^
$\:\rho\:$ (kg/m^3^)	1900^c^	2680 ^a^	1350 ^a^	2760 ^a^	2500 ^b^	2160 ^a^	2680 ^a^	2640 ^a^
k (mm^2^/s)	0.47	0.92	0.18	1.37	0.98	2.56	0.91	1.67

^a^^67^; ^b^^68^; ^c^^69^; ^d^^38^; ^e^^70^.

**Table 2:** Recorded depth/temperature data and calculated thermal resistances within the Knutsford-1 borehole.

Depth (m)	T (°C)	Thermal Resistance (°C/W)	Σ Thermal Resistance (°C/W)
0	9.8	0	0
715.4	27	322.26	322.26
911.1	31	70.78	393.04
1500	39	174.42	567.46
1803	43	88.86	656.32
2000	45	57.77	714.09
2231.8	51	78.53	792.62
3039	58.8	291.00	1083.59

#### Palaeoclimate-Corrected heat flow methodology

The procedure and theory for applying palaeoclimatic corrections to heat flow have been widely documented and are described from first principles in Westaway and Younger^33^.

In summary, past variations in surface temperature ∆*T*_*0*_ relative to its present-day value are approximated as a series of step changes that propagate into the ground, each starting at a particular time t’ before the present day. Each of these step changes has a specific timescale and a ∆*T* value representing the difference in surface temperature between that of the respective time period and that of the present day. The overall perturbation to the geotherm δ*T(z)* due to the effect of palaeoclimate, at each depth *z*, at the present day, is determined by adding the contributions of each of these step changes.

Westaway and Younger^33^ account the theory from first principles, giving an analytic formula for δ*T(z)* as a result of *n* past step changes ∆*T*_*0i*_ (*i* = 1 to *n*) in surface temperature, shown in Eq. 2.

2 $$\begin{aligned}\delta\:T\:\left(z,\:{t}^{{\prime\:}}\right)&=\:\varDelta\:\:{T}_{1}\left(1-\text{erf}\left(\frac{z}{\sqrt{4\kappa\:{t}_{1}^{{\prime\:}}}}\right)\right)\\ & \quad +\:\varDelta\:\:{T}_{2}\left(\text{erf}\left(\frac{z}{\sqrt{4\kappa\:{t}_{1}^{{\prime\:}}}}\right)-\text{e}\text{r}\text{f}\left(\frac{z}{\sqrt{4\kappa\:{t}_{2}^{{\prime\:}}}}\right)\right)\:+\dots\\& \quad +\:\varDelta\:\:{T}_{n-1}\left(\text{erf}\left(\frac{z}{\sqrt{4\kappa\:{t}_{n-2}^{{\prime\:}}}}\right)-\text{e}\text{r}\text{f}\left(\frac{z}{\sqrt{4\kappa\:{t}_{n-1}^{{\prime\:}}}}\right)\right)\\& \quad +\:\varDelta\:\:{T}_{n}\left(\text{erf}\left(\frac{z}{\sqrt{4\kappa\:{t}_{n-1}^{{\prime\:}}}}\right)-\text{e}\text{r}\text{f}\left(\frac{z}{\sqrt{4\kappa\:{t}_{n}^{{\prime\:}}}}\right)\right)\end{aligned}$$

The perturbation to the geothermal gradient ∂δ*T*/∂*z*, is found analytically by term-by-term differentiation of Eq. 2 and is given as Eq. 3. Applying Fourier’s Law, the perturbation to the geothermal gradient is scaled by the thermal conductivity of the bedrock, $\:\lambda\:$, to determine the perturbation to the heat flow at depth *z*, δ*Q(z)*. The assumed history of surface temperature variation therefore determines the present-day perturbation to the geothermal gradient, and the resulting heat flow perturbation scales in proportion to $\:\lambda\:$.

3 $$\begin{aligned}\frac{\partial\:\delta\:T}{\partial\:z}\:\left(z,\:{t}^{{\prime\:}}\right)&=\:\frac{1}{\sqrt{\pi\:}}\:\times\:\:\left[\varDelta\:\:{T}_{1}\:\frac{1}{\sqrt{\kappa\:{t}_{1}^{{\prime\:}}}}\:\:\left(-\text{exp}\left(\frac{-{z}^{2}}{4\kappa\:{t}_{1}^{{\prime\:}}}\right)\right)+\:\varDelta\:\:{T}_{2\:}\left(\frac{1}{\sqrt{\kappa\:{t}_{1}^{{\prime\:}}}}\text{exp}\left(\frac{-{z}^{2}}{4\kappa\:{t}_{1}^{{\prime\:}}}\right)-\frac{1}{\sqrt{\kappa\:{t}_{2}^{{\prime\:}}}}\:\text{exp}\left(\frac{-{z}^{2}}{4\kappa\:{t}_{2}^{{\prime\:}}}\right)\right)\right. \\& \quad +\dots\:+\:\varDelta\:\:{T}_{n-1\:}\left(\frac{1}{\sqrt{\kappa\:{t}_{n-2}^{{\prime\:}}}}\text{exp}\left(\frac{-{z}^{2}}{4\kappa\:{t}_{n-2}^{{\prime\:}}}\right)-\frac{1}{\sqrt{\kappa\:{t}_{n-1}^{{\prime\:}}}}\:\text{exp}\left(\frac{-{z}^{2}}{4\kappa\:{t}_{n-1}^{{\prime\:}}}\right)\right)\\& \quad \left. +\varDelta\:\:{T}_{n\:}\left(\frac{1}{\sqrt{\kappa\:{t}_{n-1}^{{\prime\:}}}}\text{exp}\left(\frac{-{z}^{2}}{4\kappa\:{t}_{n-1}^{{\prime\:}}}\right)-\frac{1}{\sqrt{\kappa\:{t}_{n}^{{\prime\:}}}}\:\text{exp}\left(\frac{-{z}^{2}}{4\kappa\:{t}_{n}^{{\prime\:}}}\right)\right)\right]\end{aligned}$$

Westaway and Younger^33^ developed a program which evaluates Eq. 3 to determine ∂δT/∂z and δQ as functions of depth z. The solution for ∂δT/∂z is numerically integrated using Simpson’s Rule to determine the associated perturbation to temperature δT(z). This methodology was used within the present study.

The following parameters are required as input to the palaeoclimate correction modelling:

(1), subsurface temperature measurements within the depth interval ∆z over which heat flow was calculated in the borehole,

(2) the harmonic mean thermal conductivity $\:\lambda\:$ and the associated thermal diffusivity κ of the depth interval ∆z over which heat flow was calculated in the borehole, and,

(3) a time-series of variations in surface temperature.

#### Input data required for palaoeclimate correction modelling

For this study, the palaeoclimate corrected heat flow was modelled over a depth range of 0–3039 m, consistent with the calculation of uncorrected heat flow previously described. This incorporates temperature measurements from a depth range of 0–3039 m, including both the ground surface temperature of 9.8 °C calculated in the present study, and the temperature measurements recorded between 715 and 3039 m within the borehole^37,39,71^.

The harmonic mean thermal conductivity and thermal diffusivity between 0 and 3039 m within the Knutsford-1 borehole were then calculated as 2.81 W/m°C and 1.36 mm^2^/s respectively. Calculation of the harmonic mean thermal conductivity and thermal diffusivity accounts for the small, frequent, changes in lithology present in the stratigraphic sequence encountered by the Knutsford-1 borehole. These calculations are based on the borehole lithological log and the representative thermal properties assigned to each lithology as shown in Table 1.

To address point (3) above, a model surface temperature history of the Cheshire Basin was established. This temperature history was constructed from a combination of data sources, including Westaway and Younger’s^33^ assumed temperature history for northern England during the Pleistocene, and literature on the timing and dynamics of the British and Irish Ice Sheet (BIIS) in the Cheshire Basin, as discussed in elsewhere in the present study.

In addition, Westaway and Younger^68^ found that the changes in surface air temperature during the years immediately preceding the year of borehole temperature measurement have a disproportionate effect on the overall palaeoclimate correction. To account for anthropogenic temperature changes and any significant fluctuations in temperature in the years prior to the borehole measurements, we also incorporate meteorological data from the Central England Temperature series^72^, spanning from the 17th century to the present day.

Following integration of these datasets, the surface temperature record from the Pleistocene to the present day was approximated as a series of step changes (*∆T*_*o*_) relative to the annual mean temperature of the year in which geothermal data was recorded in the Knutsford-1 borehole. This time-series of variations in surface temperature is shown in Tables 3 and 4, where t_1_ and t_2_ represent the start and end of each time interval. The temperature history is also illustrated in Fi.g. 9.

**Table 3:** Assumed temperature history representative of conditions in the Cheshire basin during the late Pleistocene. Present day taken as the year in which the Knutsford borehole was drilled, 1976, with T_0_ as 9.6 °C representing the annual mean surface air temperature for this year in the central England temperature series.

t_1_ (ka)	t_2_ (ka)	∆ T_0_ (°C)	Name
0	0.316	See Table 4	Latest Holocene/Present Day Conditions
0.316	3.5	0	Holocene
3.5	7.5	+ 0.40	Mid Holocene Climatic Optimum
7.5	11.5	-1.60	Early Holocene
11.5	12.8	-9.60	Younger Dryas Stadial
12.8	14.7	-5.60	Lateglacial Interstadial
14.7	21	-19.60	Last Glacial Maximum – After Deglaciation
21	27	-9.60	Last Glacial Maximum – During Glaciation
27	45	-15.60	Earlier MIS 2
45	65	-9.60	MIS 3
65	75	-19.60	MIS 4
75	120	-7.60	MIS 5d-5a
120	130	+ 3.40	Ipswichian (MIS 5e)
120	3000	-5.60	Earlier Pleistocene
3000	65,000	+ 3.40	Pre-Quaternary

**Fig. 3:**
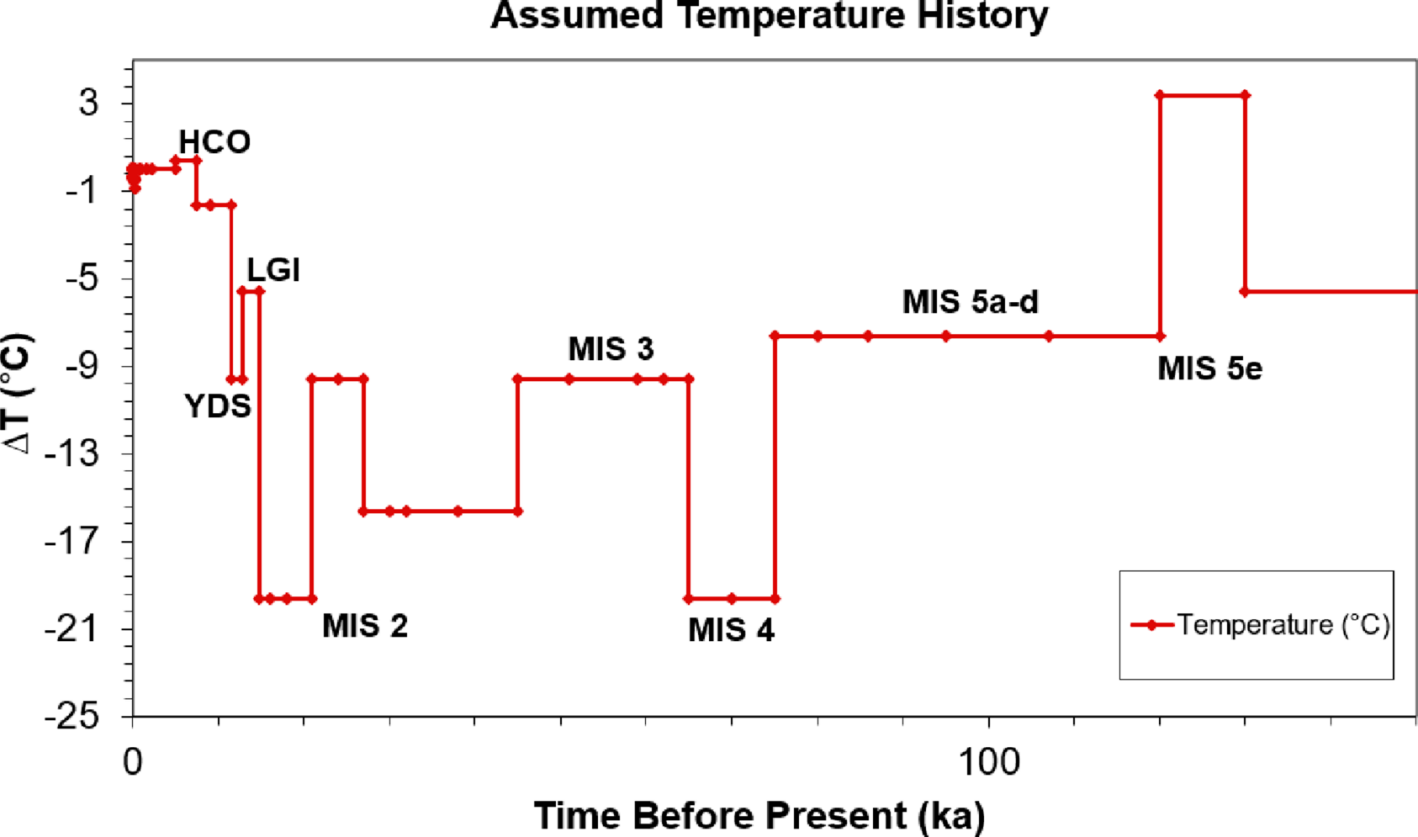
Assumed temperature history (also tabulated in Tables 3 and 4), representative of conditions in the Cheshire Basin during the Late Pleistocene. Present day taken as the year in which the Knutsford borehole was drilled, 1976, with T_o_ as 9.6 °C representing the annual mean surface air temperature for this year.

Times t_1_ and t_2_ represent the start and end of each phase, for which the surface temperature (relative to the present-day value) is assumed to have been ∆ T_0_. Individual temperature phases are named based upon British chronostratigraphy, with several being associated with particular Marine Oxygen Isotope Stage (MIS) numbers. Present day taken as the year in which the Knutsford borehole was drilled, 1976, with T_0_ as 9.6 °C representing the annual mean surface air temperature for this year. To satisfy local conditions in the Cheshire Basin, a necessary amendment to Westaway and Younger’s^33^ temperature history was made to alter the timings of glacial advance and retreat within the Cheshire Basin during the Last Glacial Maximum (Fig. 3).

**Table 4:** Temperature record for the Cheshire basin from 1659 to 1974 tabulated as a series of step changes (∆To). Annual mean surface temperature data derived from the central England temperature series^72^.

t_1_ (year)	t_2_ (year)	∆ T_0_ (°C)
1974	1973	0.00
1973	1972	0.00
1972	1971	-0.40
1971	1970	+ 0.10
1970	1960	-0.27
1960	1932	-0.01
1932	1901	-0.34
1901	1892	-0.56
1892	1859	-0.46
1859	1821	-0.46
1821	1803	-0.38
1803	1776	-0.47
1776	1760	-0.50
1760	1715	-0.43
1715	1659	-0.88

### Numerical modelling

#### Governing equations

**Fig. 4:**
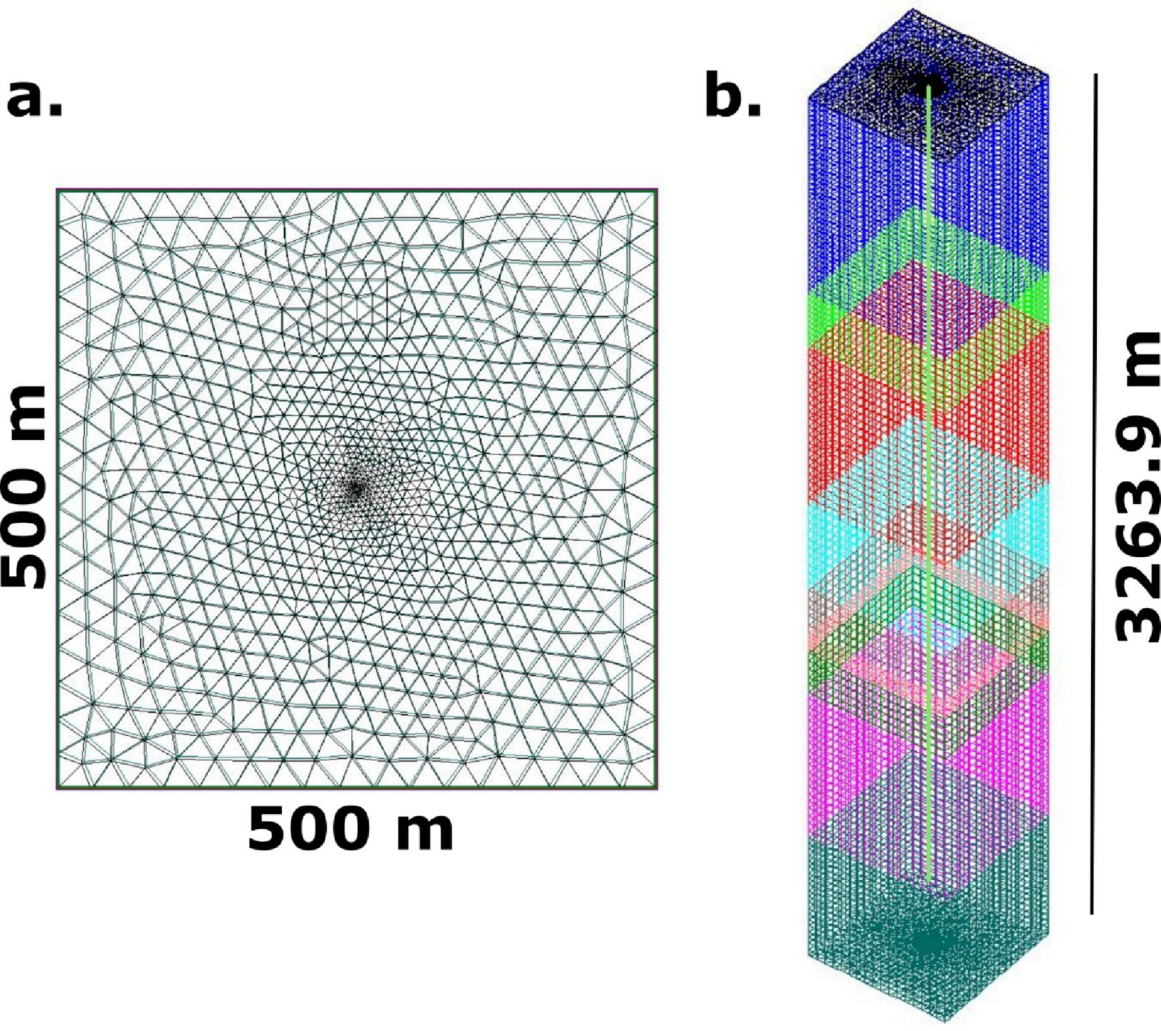
(**a**) Example of the plan view domain (i.e., at the top of the model) and (**b**) fully discretised mesh and domain size. Note the varying colours represent the varying lithological layers highlighted in Table 5.

The BHE heat transport module in OGS software was used for all simulations in this work. It adopts a ‘dual-continuum’ modelling approach with finite element method in which the centralised DBHE is treated as a 1D line elements while the surrounding rock is 3D (Fig. 4). This essentially treats the subsurface as two continuums. Lithological layering of the surrounding rock has also been incorporated into the mesh. The modelled DBHE adopts a coaxial pipe with annular inlet (Fig. 1) where fluid enters through the annulus and leaves through the central pipe. This configuration was chosen because it slightly outperforms the opposite configuration (central pipe inlet and annular outlet) with no major influence^14^. The DBHE model was simplified with a single grout layer and a single casing (Fig. 1). Through the application of a constant regional Darcy velocity ($\:{v}_{f}$) – see Eq. 4 – the effects of groundwater flow have been modelled.

The complete model consists of four governing equations for (a) the surrounding rock, (b) the cementitious grout, (c) the borehole casing (outlet pipe) and (d) the central pipe (inlet pipe). The surrounding rock governing equation is given by (e.g^21,44^) :

4 $$\:\frac{\partial\:{T}_{r}}{\partial\:t}\left[\varnothing\:{\rho\:}_{f}{c}_{f}+(1-\varnothing\:){\rho\:}_{r}{c}_{r}\right]+\nabla\:\cdot\:\left({\rho\:}_{f}{c}_{f}{v}_{f}{T}_{r}\right)-\nabla\:\cdot\:\left({\varLambda\:}_{r}\cdot\:\nabla\:{T}_{r}\right)={H}_{r}$$

in which $\:\varnothing\:$ is rock porosity, $\:{\rho\:}_{r}$is rock matrix density, $\:{\rho\:}_{f}$is groundwater flow density, $\:{T}_{r}$is surrounding rock temperature, $\:{c}_{r}$ is rock matrix specific heat capacity and $\:{c}_{f}$is groundwater specific heat capacity. $\:\nabla\:$ is the gradient operator $\:(\frac{\partial\:}{\partial\:x},\frac{\partial\:}{\partial\:y},\frac{\partial\:}{\partial\:z})$ .

For the static setup the change in temperature was assumed purely conductive and set such that the change in temperature was proportional to zero (e.g., see^73^).

$\:{H}_{r}$ is the source term for the rock and $\:{\varLambda\:}_{r}$ is the thermal hydrodynamic dispersion tensor given by^74^

5 $$\:{\varLambda\:}_{r}=\left[\varnothing\:+(1-\varnothing\:){\lambda\:}_{r}\right]\varvec{I}+{\alpha\:}_{T}||\varvec{v}||\varvec{I}\left({\alpha\:}_{L}-{\alpha\:}_{T}\right)\left[\frac{\varvec{v}\otimes \varvec{v}}{||\varvec{v}||}\right]$$

in which $\:{\lambda\:}_{f}$ is the thermal conductivity of groundwater, $\:{\lambda\:}_{r}$ is the thermal conductivity of the rock, $\:\varvec{v}$ is velocity vector, $\:\varvec{I}$ is the identity matrix, $\:{\alpha\:}_{L}$ and $\:{\alpha\:}_{T}$ are the longitudinal and transverse thermos-dispersivity respectively.

Between the surrounding rock and the DBHE, a heat flux ($\:{q}_{n{T}_{r}}$) boundary condition was imposed:

6 $$\:{q}_{n{T}_{r}}=-({\varLambda\:}_{r}\cdot\:\nabla\:{T}_{r})$$

The governing equation of the grout (dominated by conduction) is taken as:

7 $$\:\frac{\partial\:{T}_{g}}{\partial\:t}\left[(1-\varnothing\:){\rho\:}_{g}{c}_{g}\right]-\nabla\:\cdot\:\left({(1-\varnothing\:)\lambda\:}_{g}\cdot\:\nabla\:{T}_{g}\right)=\:{H}_{g}$$

In which subscript $\:g$ indicates grout. The grout has different physical properties and porosity to the rock. Advection heat transfer is present in the inlet (subscript $\:i$) or annular space and the outlet (subscript $\:o$) or central coaxial pipe :

8 $$\:\frac{\partial\:{T}_{i}}{\partial\:t}{\rho\:}_{f}{c}_{f}+{\rho\:}_{f}{c}_{f}{v}_{i}\cdot\:\nabla\:{T}_{i}-\nabla\:\cdot\:\left({\varLambda\:}_{f}\cdot\:\nabla\:{T}_{i}\right)=\:{H}_{i}$$

9 $$\:\frac{\partial\:{T}_{o}}{\partial\:t}{\rho\:}_{f}{c}_{f}+{\rho\:}_{f}{c}_{f}{v}_{o}\cdot\:\nabla\:{T}_{o}-\nabla\:\cdot\:\left({\varLambda\:}_{f}\cdot\:\nabla\:{T}_{o}\right)=\:{H}_{o}$$

with $\:{v}_{i}$ and $\:{v}_{o}$ representing the inlet and outlet fluid velocity vectors, respectively. $\:{\varLambda\:}_{f}$ is the hydrodynamic thermo-dispersion tensor which simplifies to the fluid thermal conductivity ($\:{\lambda\:}_{f}$).

Using an analogue to a resistor network, the horizontal thermal resistance to heat flow was modelled following Al-Khoury et al.^75^. There are three thermal resistances to heat flow: (i) $\:{R}_{gr}$ for resistance to heat flow between the rock and grout; (ii) $\:{R}_{fig}$for resistance to heat flow between the grout and the borehole casing (inlet); and (iii) $\:{R}_{ff}$ for resistance to heat flow between the inlet and outlet (central coaxial pipe). These thermal resistances can be expressed as heat transfer coefficients ($\:\varPhi\:$) by using the outer surface area for each interface, and subsequently, employed to impose boundary conditions for Eqs. (6)–(9). Using the outer surface area at the relevant interface, the thermal resistances can be expressed as heat transfer coefficients which appear in the boundary conditions for the grout, borehole casing, and central coaxial pipe. The boundary conditions for the governing equation of the grout, inlet and outlet are expressed respectively as:

10 $$\:{q}_{n{T}_{g}}=-{\varPhi\:}_{gr}\left({T}_{r}-{T}_{g}\right)-{\varPhi\:}_{fig}\left({T}_{i}-{T}_{g}\right)$$

11 $$\:{q}_{n{T}_{i}}=-{\varPhi\:}_{fg}\left({T}_{r}-{T}_{i}\right)-{\varPhi\:}_{ff}\left({T}_{o}-{T}_{i}\right)$$

12 $$\:{q}_{n{T}_{o}}=-{\varPhi\:}_{ff}\left({T}_{i}-{T}_{o}\right)$$

The heat transfer coefficients depend on the borehole casing diameter, the central coaxial pipe diameter, and the borehole diameter. The heat transfer coefficients are related to resistances and these can be found in Diersch et al.^74^.

#### Parameterisation, initial and boundary conditions

The parameters for the model were determined based off literature and the Knutsford-1 well logs. Initially, the thermal properties for each formation were calculated using a weighted average for each lithology (see Table 5) based upon varying lithologies matched to drilling logs (Table 1). These consist of upper Quaternary rocks, underlain by the insulating Mercia Mudstone Group before passing through a thick succession of Permo-Triassic sandstones which are prevented from being in hydraulic continuity by the Manchester Marls Formation. When modelling the borehole a weighted average was undertaken for the varying hole sizes (see Table 6); this resulted in an average of 13 inch (or 0.33 m) diameter being implemented. All other parameters are listed in Table 7. The flow rate was chosen as a typical value found to be optimal for past DBHEs^15,22^, although this was subsequently systematically varied in analysis. Fluid properties were assumed to be consistent for that of fluid inlet temperature. Surface temperature was assigned in line with the national average. The grout and pipe thermal properties, central pipe geometrical values and pump efficiency were assumed after a recent project focusing on repurposing a borehole in Newcastle^12,76^.

**Table 5:** Different parameter values assigned for each geological layer.

Formation/ Group	Thermal Conductivity (W/mK)	Density (kg/m^3^)	Specific Heat Capacity (J/kg·K)	Thickness (m TVD)^1,2^	Porosity^3^	Hydraulic Conductivity^4^ (m/s)	Darcy Velocity (m/s)
Quaternary	0.77	1900	860	5	0.2	0	0
MMG	2.24	2524	820	742	0.1	10^− 7^	10^− 9^
Helsby Formation	2.93	2643	793	205	0.162	1.2 × 10^− 5^	1.2 × 10^− 7^
Wilmslow Sandstone Formation	3.41	2640	775	595	0.1879	1.2 × 10^− 5^	1.2 × 10^− 7^
Wilmslow Sandstone Formation (Silicified)	3.41	2640	775	325.5	0.1879	10^− 8^	10^− 10^
Chester Formation	3.41	2640	775	164.5	0.0817	1.2 × 10^− 5^	1.2 × 10^− 7^
Kinnerton Sandstone Formation	3.41	2450	775	74.5	0.1625	1 × 10^− 6^	7.9 × 10^− 7^
Manchester Marls Formation	2.6	2586	773	148	0.1	10^− 8^	10^− 10^
Collyhurst Sandstone Formation	3.26	2632	775	556	0.11	1 × 10^− 6^	7.9 × 10^− 7^
Carboniferous Coal Measures	2.04	2599	839	448.2	0.1	10^− 8^	10^− 10^

References: 1 –^77^, 2 –^37^. 3 – porosity values for Carboniferous to Triassic sandstones taken from Hirst^78^. 4 – Wilson et al.^79^. Note the Darcy velocity for each layer was calculated by applying a hydraulic gradient in the x direction of 1%. This is assumed to be a high-end case to understand the impact of groundwater flow. The Carboniferous coal has also been extended to the base of the model. Kinnerton and Collyhurst hydraulic conductivity reduced by an order of magnitude from the values from Wilson et al. to represent the compaction from overburden.

**Table 6:** Different borehole diameters and corresponding lengths for the Knutsford-1 borehole (after Wight^37^).

Hole Size (inches)	Length (m)
26	96.3
17.5	762.3
12.25	1361.5
8.5	819.6

**Table 7:** Base case parameters.

Parameter	Value	Units
Borehole diameter	330	mm
Borehole length	3039.7	m
Water flow rate	5	L/s
Water density	999	kg/m^3^
Water volumetric heat capacity	4.18 × 10^6^	J/(K m^3^)
Water thermal conductivity	0.59	W/(m.K)
Dynamic water viscosity	15 × 10^− 4^	kg/(m s)
Current surface temperature	10	°C
Cement thermal conductivity	1.05	W/(m.K)
Cement volumetric heat capacity	1.19 × 10^6^	J/(K m^3^)
Outer pipe outer diameter	0.1779	m
Outer pipe thickness	0.0081	m
Outer pipe thermal conductivity (steel)	52.7	W/(m.K)
Central pipe outer diameter	0.1005	m
Central pipe thickness	0.00688	m
Central pipe thermal conductivity (HDPE)	0.45	W/(m.K)
Circulation Pump Efficiency	70	%

Under initial conditions, a static setup was undertaken to calculate the variable geothermal gradient for varying layers (where $\:\frac{\partial\:{T}_{r}}{\partial\:t}$=0). This was undertaken by using a 1D finite-difference model, with the corrected and uncorrected heat flows input at the base of the model and a constant surface temperature^73^. The varying thermal gradient was then imported to OpenGeoSys for transient simulations. Under initialisation of the simulation, all wellbore components (grout, pipe, fluid) were set to equal initial conditions (i.e., increase with depth under the natural geothermal gradient), such that $\:\frac{\partial\:{T}_{r}}{\partial\:t}=\frac{\partial\:{T}_{g}}{\partial\:t}=\frac{\partial\:{T}_{i}}{\partial\:t}=\frac{\partial\:{T}_{o}}{\partial\:t}$.

For the transient models, the surface temperature was set at a fixed constant Dirichlet boundary corresponding to 10 °C (i.e., $\:{T}_{r}\left(x,y,z=0,t\right)=10^\circ\:$C), the lateral boundaries were fixed as Neumann no-flow boundaries with heat flux set equal to zero (i.e., $\:\frac{\partial\:{T}_{r}}{\partial\:x}=0,\:\frac{\partial\:{T}_{r}}{\partial\:y}=0$) and the base of the model was prescribed as a constant heat flow boundary ($\:i.e.,\:\frac{\partial\:{T}_{r}}{\partial\:z}=-\frac{q}{\lambda\:}$), where $\:q$ is the heat flow. Lateral and basal boundaries of the model were set to extend to a distance where thermal interaction with the DBHE would be minimal (and not occur). For the setup of groundwater flow, a constant Darcy velocity was set up in the x direction throughout the model, based on the hydraulic conductivity and an assumed hydraulic gradient of 1%. Finally, the domain size was set at 500 × 500 × 3263.9 m (x, y,z) (Fig. 4). During the simulations the maximum thermal propagation to within 0.1 °C of initial conditions during the conductive simulations was 85 m away from the borehole, which was recorded at the end of the simulation. Similarly, for the simulations with groundwater flow the maximum spread of the thermal plume was to 210 m from the borehole. Thus, there was no impact of the boundaries. Mesh testing was undertaken prior to simulation to ensure optimal design around the DBHE in-line with methods from Shao et al.^44^ and the software has been rigorously tested against data from case studies, analytical solutions (such as the solution from Beier^80^) and other industry leading software (e.g^13,21,81–83^). Past software verification has highlighted that there are minimal changes in solution output under different mesh sizes, as the main control is the optimal mesh spacing around the DBHE and the same approach has been used here (e.g^84,85^).

#### Heat pump equations

The efficiency of the system as a relationship between the heat load supplied to the building ($\:{P}_{building}$) and the amount of energy consumed by a heat pump ($\:{W}_{hp}$) is defined by the coefficient of performance ($\:COP$):

13 $$\:COP=\frac{{P}_{building}}{{W}_{hp}}$$

The $\:COP$ can also be estimated using the outlet temperature ($\:{T}_{out}$) from the deep borehole heat exchanger. For an assumed heat pump outlet temperature of 35 °C, the COP is expressed as (e.g^86^):

14 $$\:{COP=(T}_{out}\times\:0.083)+3.925$$

The thermal power from the deep borehole heat exchanger ($\:{P}_{DBHE}$) can be expressed as a function of the building load and the power consumed by the heat pump as:

15 $$\:{{P}_{DBHE}=P}_{building}-\:{W}_{hp}$$

Or as a function of the coefficient of performance as:

16 $$\:{P}_{building}{=\frac{COP}{COP-1}P}_{DBHE}$$

The coefficient of system performance ($\:CSP$) for the whole system accounts for the energy consumed by the circulation pump ($\:{W}_{cp}$) in addition to $\:{W}_{hp}$. It is given by (e.g^21^):

17 $$\:CSP=\frac{{P}_{building}}{{W}_{hp}+{W}_{cp}}$$

in which $\:{W}_{cp}$ is calculated using (e.g^87^):

18 $$\:{W}_{cp}=\frac{\varDelta\:p\:\times\:Q}{n\:}$$

with $\:\varDelta\:p$ the pressure drop in the DBHE and $\:n$ the circulation pump efficiency (taken to be 70%). The pressure drop was calculated as the sum of pressure drop in the inlet and outlet. For each component, the Darcy-Weisbach equation and Petukhov’s version of the friction factor, valid down to Re = 3000 was used to compute the pressure drop^21,88,89^:

19 $$\:\varDelta\:p=\frac{L{\rho\:}_{f}{V}_{f}^{2}}{2{D}_{h}{[0.79\text{ln}\left(Re\right)-1.64]}^{2}}$$

in which $\:L$ is the pipe length, $\:{V}_{f}$ is the mean velocity of the inlet/outlet, $\:{D}_{h}$ is the hydraulic diameter of inlet/outlet pipe, and $\:Re$ is the Reynolds number of inlet/outlet pipe. In this study, the flow is assumed to be turbulent.

## Results

In this section, results of palaeoclimate corrections, modelling of steady state initial conditions and transient simulations are presented. Transient numerical modelling was undertaken on OpenGeoSys, a finite-element solver, whilst the steady state set-up was conducted as a 1D temperature model with lithological layers. The steady state model then was used to represent initial conditions and incorporated into the 3D model.

### Palaeoclimate corrections for the Cheshire basin

Until the present study, applying a correction to account for the palaeoclimatic effect on heat flow had not been made to any borehole dataset from the Cheshire Basin. By application of the Bullard method^64^ an uncorrected heat flow of 46.2 mW/m^2^ was determined for the Knutsford-1 borehole, which had hitherto not been calculated.

For comparison, Table 8 summarises the previously published heat flow measurements made within boreholes in the Cheshire Basin. It should be noted that these values of heat flow have not been corrected for the effect of palaeoclimate, and this should be considered in future work. As shown, the uncorrected heat flow determined for Knutsford-1 lies within the range of uncorrected heat flow measured across the Cheshire Basin, and is higher than the average uncorrected heat flow of 41 mW/m^2^.

**Table 8:** Summary of previously published heat flow values from boreholes within the Cheshire Basin.

Borehole	BGS Ref	BNG Ref	Year of measurement	Elevation (m)	Depth (m)	Heat flow Interval (m)	Q (mW/m^2^)
Bradley Mill	SJ57NW14	SJ 53,080 76,760	1968	63	219.5	70–190	59^a^
Clotton	SJ56SW10	SJ 52,780 63,580	1973	40	-	0-305	33^a^
Organsdale	SJ56NE15/B	SJ 55,300 68,400	07.10.1971	105	457.5	70–470	25^a^
Priory Heyes	SJ56NW15	SJ 51,308 66,470	16.08.1972	30	335.28	10–340	34^a^
Holford	-	SJ 69,200 74,400	1939	30	-	168–396	38^b^
Crewe	SJ65SE6	SJ 68,270 54,520	13.01.1984	40	299.28	100–296	57^c^

References: (a)^40^; (b)^34^; (c)^41^. BNG is British National Grid and BGS is British Geological Survey.

The palaeoclimate corrected heat flow for the Knutsford-1 borehole, on the other hand, was determined to be 51.7 mW/m^2^ for a harmonic mean thermal conductivity of 2.81 W/m°C and thermal diffusivity of 1.36 mm^2^/s, with the assumed temperature history illustrated in the Methods section. This represents a 12% increase in heat flow. For comparison, in Westaway and Younger^33^, correcting for the effect of palaeoclimate resulted in an increase in heat flow ranging between 12 and 27% for the 6 boreholes included in their study. Whilst the palaeoclimate correction to heat flow determined for the Knutsford-1 borehole lies to the lower end of this range, it nonetheless illustrates the extent to which heat flow was previously underestimated.

### Numerical modelling

#### Steady state Set-up

**Fig. 5:**
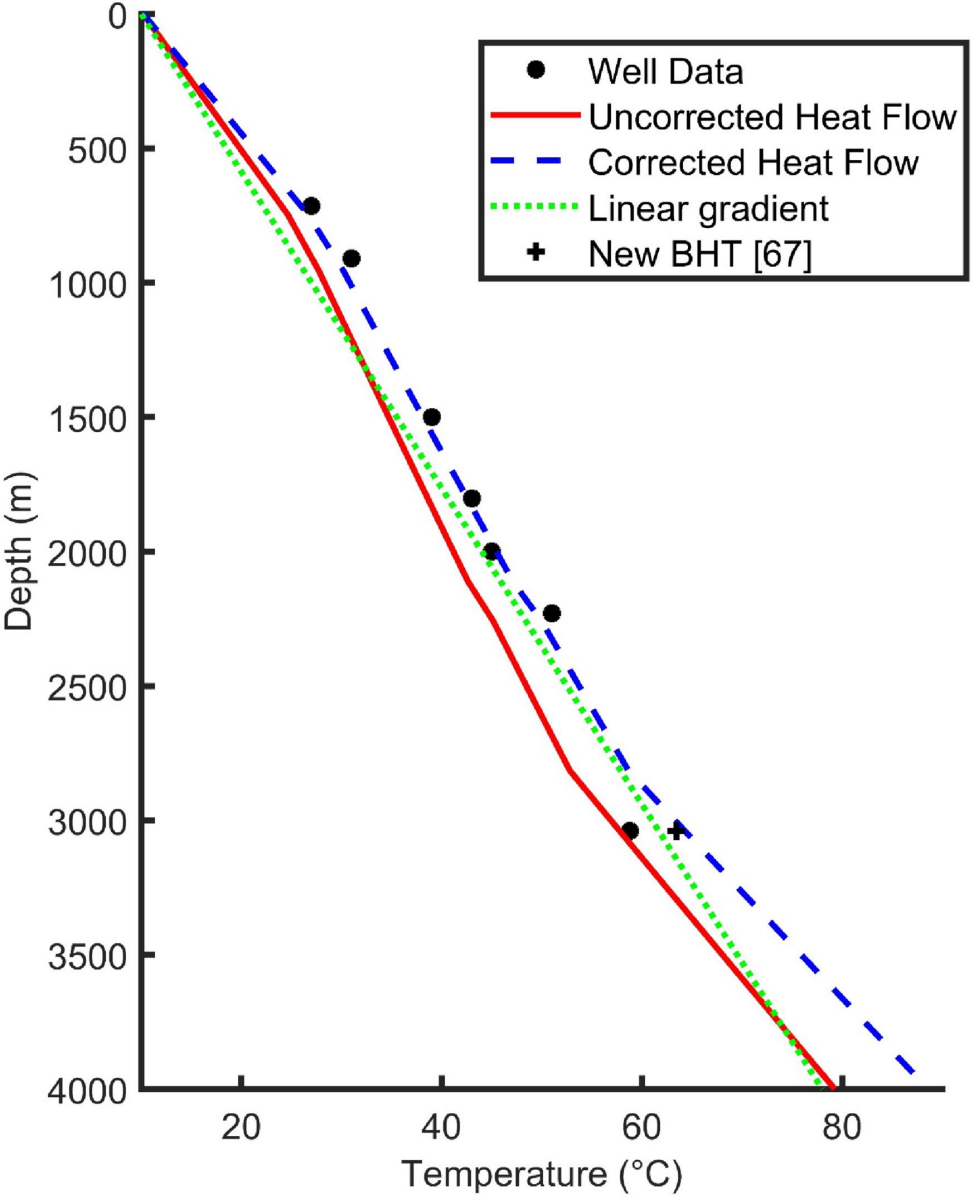
Temperature variations with depth generated from the static setup using corrected and uncorrected heat flows. Note a linear gradient of 17 °C/ km is also shown for comparison and a newly released re-equilibrated bottom hole temperature (BHT) is shown^91^. Data points from Plant et al.^39^ and Wright^37^.

Following this palaeoclimatic correction to heat flow for the Knutsford-1 borehole, a steady state setup was undertaken to predict varying temperature and geothermal gradients at depth, corresponding to different lithological layers (Table 5). Both corrected and uncorrected heat flows were used as the input to the base of the model and then the model iterated until convergence was reached. This was achieved when the sum of temperature variations over the length of the well varied < 5e-4 in contrast to the previous iteration. Figure 5 shows (i) a steady state setup using the uncorrected heat flow where palaeoclimate corrections have not been incorporated, (ii) a static setup using the corrected heat flow value, (iii) a linear gradient, and (iv) the data recorded from the borehole from^39^ and^37^.

When using the uncorrected heat flow, predicted temperature values are far lower than the measured values. This is because the uncorrected heat flow corresponds to the average over the borehole and is similar to past estimates for heat flow for the basin (e.g^39^). The corrected heat flow gives a proximal fit in comparison to the measured data (and is generally within 1.8 °C, excluding the bottom hole temperature value). The maximum difference between the uncorrected data and measured data was recorded at 2230 m, where the measured temperature was 51 °C with the modelled temperature 44.65 °C (a difference of -6.35 °C). The maximum difference between the corrected and measured data was at the bottom-hole measurement, with the measured temperature 58.8 °C and the modelled temperature 64.28 °C (a temperature difference of 5.48 °C). Typically, a linear gradient may also be used, thus we have included this for comparison – assuming a geothermal gradient of 17 °C/ km. The root mean squared error for the uncorrected, corrected and linear scenarios for the static set ups in comparison to the raw data was 4.1 °C, 2.3 °C and 3.6 °C, respectively. This highlights greater discrepancy for the uncorrected and linear cases in comparison to the raw data.

This provides strong evidence that palaeoclimate corrections can improve the estimates of heat flow from boreholes and, thus, provide more accurate steady state setups of the variable geothermal gradient with depth. It also provides strong evidence that the basin-wide heat flow in Cheshire is underpredicted. Furthermore, although it is not mentioned in Plant et al.^39^, it is believed the borehole temperature was not corrected initially (i.e., using a Horner plot or other methods) so it is possible that the measured data are not fully accurate^90^, and it is likely that the cooled bottom hole temperature could be higher. This can have significant implications for DBHEs as a reduced predicted temperature (using lower, uncorrected heat flows, or non-equilibrium bottomhole temperatures) will result in modelling domains with less energy available for extraction.

#### Transient results

**Fig. 6:**
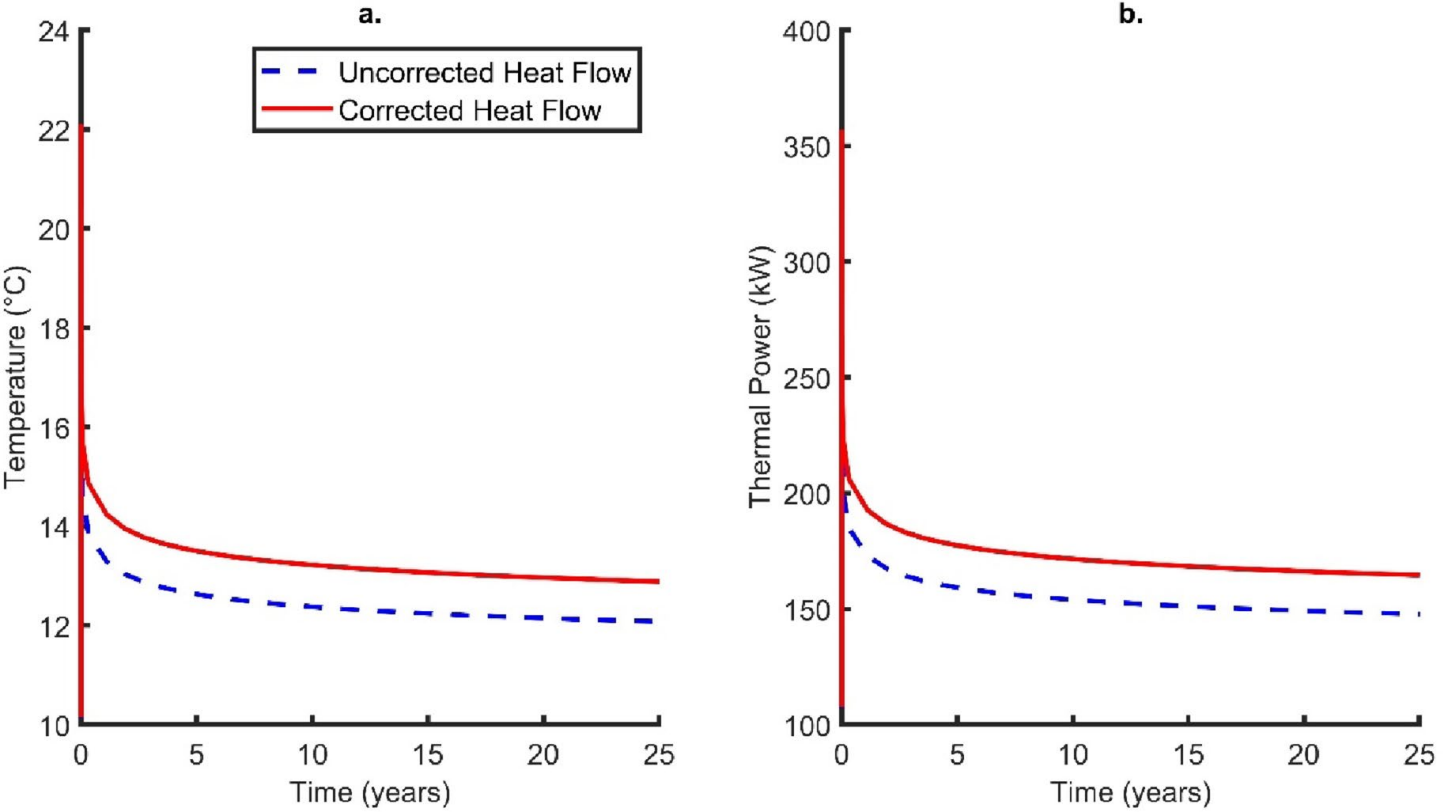
(**a**) Outlet temperature and (**b**) thermal power change with time for both corrected and uncorrected scenarios. Note that the inlet temperature is fixed at 5 °C.

Following the static setup, both temperature profiles generated using corrected and uncorrected heat flows were used as initial conditions for temperature in the transient solution on OGS. The thermal response from both the uncorrected and corrected models shows similar outlet fluid temperature curves when plotted against time (Fig. 6); initially there is a rapid increase in temperature which peaks at 3.4 h with temperatures of 20.33 and 22.06 °C (for uncorrected and corrected heat flows, respectively). This is due to the circulation of fluid at the base of the borehole reaching the surface and under initial conditions fluid temperature increases with depth due to the geothermal gradient. The outlet temperature then rapidly drops before settling into a gradual logarithmic decline. The temperature at the end of the simulation (25 years) was recorded at 12.08 °C for the uncorrected heat flow and 12.89 °C for the corrected heat flow. These correspond to thermal powers of 148 and 165 kW, respectively (difference of 17 kW).

Under initial conditions, the bottom hole temperature for the corrected heat flow steady state profile was 64.3 °C. At the end of the simulation, the bottom hole temperature reaches 27.1 °C, showing significant thermal drawdown and cooling of the system. For the uncorrected model, the bottom hole temperature was recorded as 24.8 °C at the end of the simulation.

Another simulation was undertaken using an initial linear gradient of 17 °C/km which is calculated from the temperature log at 3 km and surface temperature of 10 °C (Fig. 5). The uncorrected heat flow value was used as the basal condition, as it is similar to the regional average. This was undertaken to highlight the differences that occur through varied initial conditions. It was shown for the linear case the final outlet temperature recorded at the end of the simulation was 12.33 °C, corresponding to a thermal power of 154 kW. This differs from the corrected heat flow model by 11 kW.

The hydraulic performance of DBHEs is also important to the overall performance. The total pressure drop across the system is high (372 kPa), due to the increased length of the borehole and turbulent flows (Reynolds number of 16170 and 48880 in the annulus and central pipe, respectively). Consequently, the electrical energy consumed is high, with circulation pump energy used estimated as 2.66 kW. As the simplified equations for pressure drop do not account for variations in density/viscosity due to changes in temperature pressure drop, Reynolds number and circulation pump energy consumption is identical for both corrected and uncorrected models. However, the electrical energy consumed by the heat pump varies with outlet temperature. The corrected model value was 41.22 kW, while the uncorrected model value was 37.63 kW. As a result, the coefficient of system performance (CSP) varies slightly (4.7 and 4.6, respectively for corrected and uncorrected heat flows) and the parasitic power (inclusive of electricity used by the heat pump and circulation pump) to thermal power ratio is poor for both models (26.6% and 27.3%, respectively for corrected and uncorrected heat flows).

##### Influence of varying groundwater flow

As the Cheshire Basin’s stratigraphy is dominated by sandstones with high porosity and permeability, the effect of groundwater flow on the system was modelled. Previous work has highlighted that high velocity groundwater flows (forced or free convection), over large thicknesses can have a positive impact on performance^13,92^, whilst when the aquifer is thin, the impact is minor^21^. In this case, the impact of forced convection over different lithological layering (with Darcy Velocities given in the methods section) on DBHE performance was tested.

**Fig. 7:**
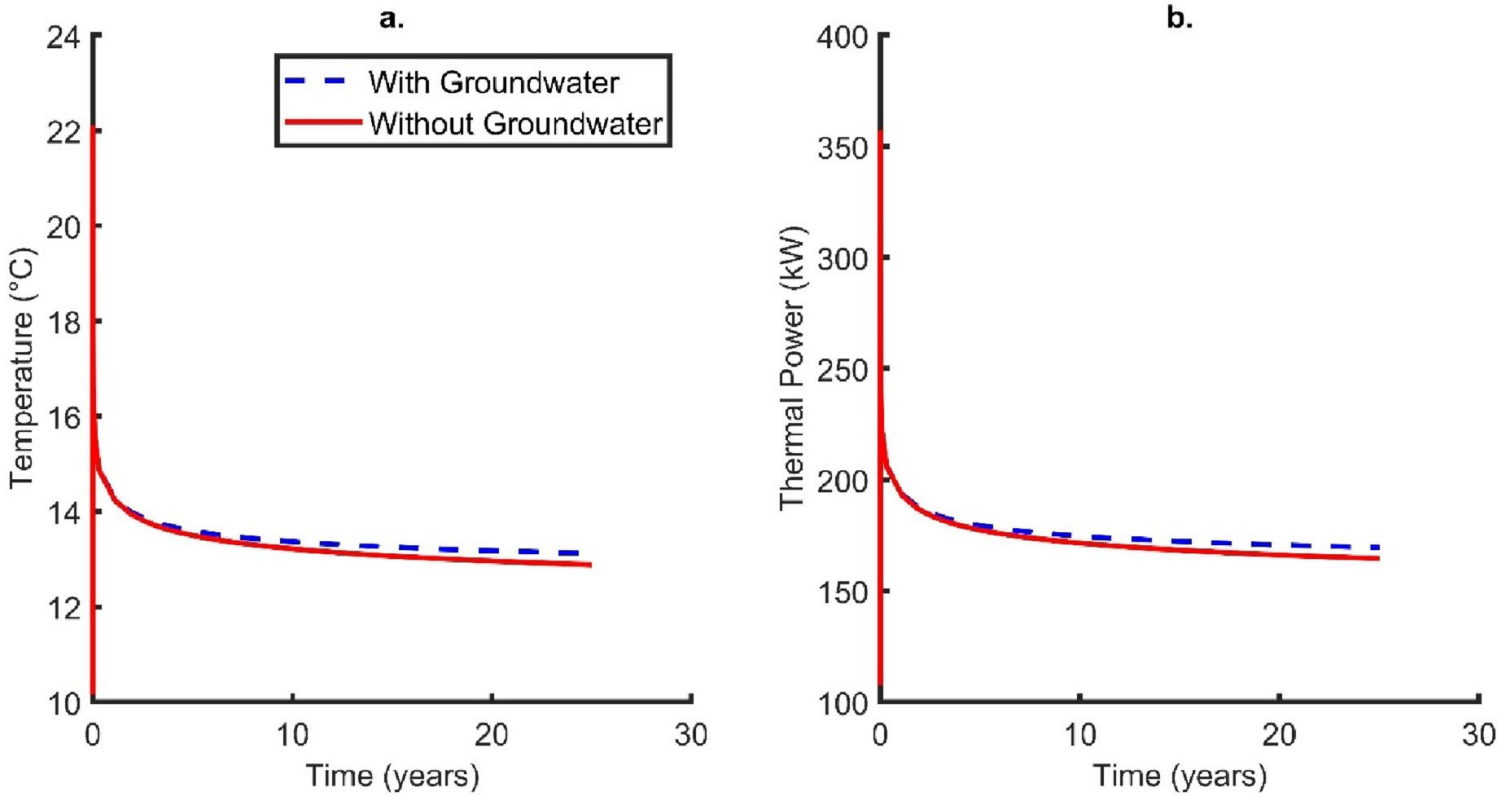
(**a**) Outlet temperature and (**b**) thermal power change with time for cases of conductive heat transfer only (corrected scenario) and advective groundwater flow. Note that the inlet temperature is fixed at 5 °C.

**Fig. 8:**
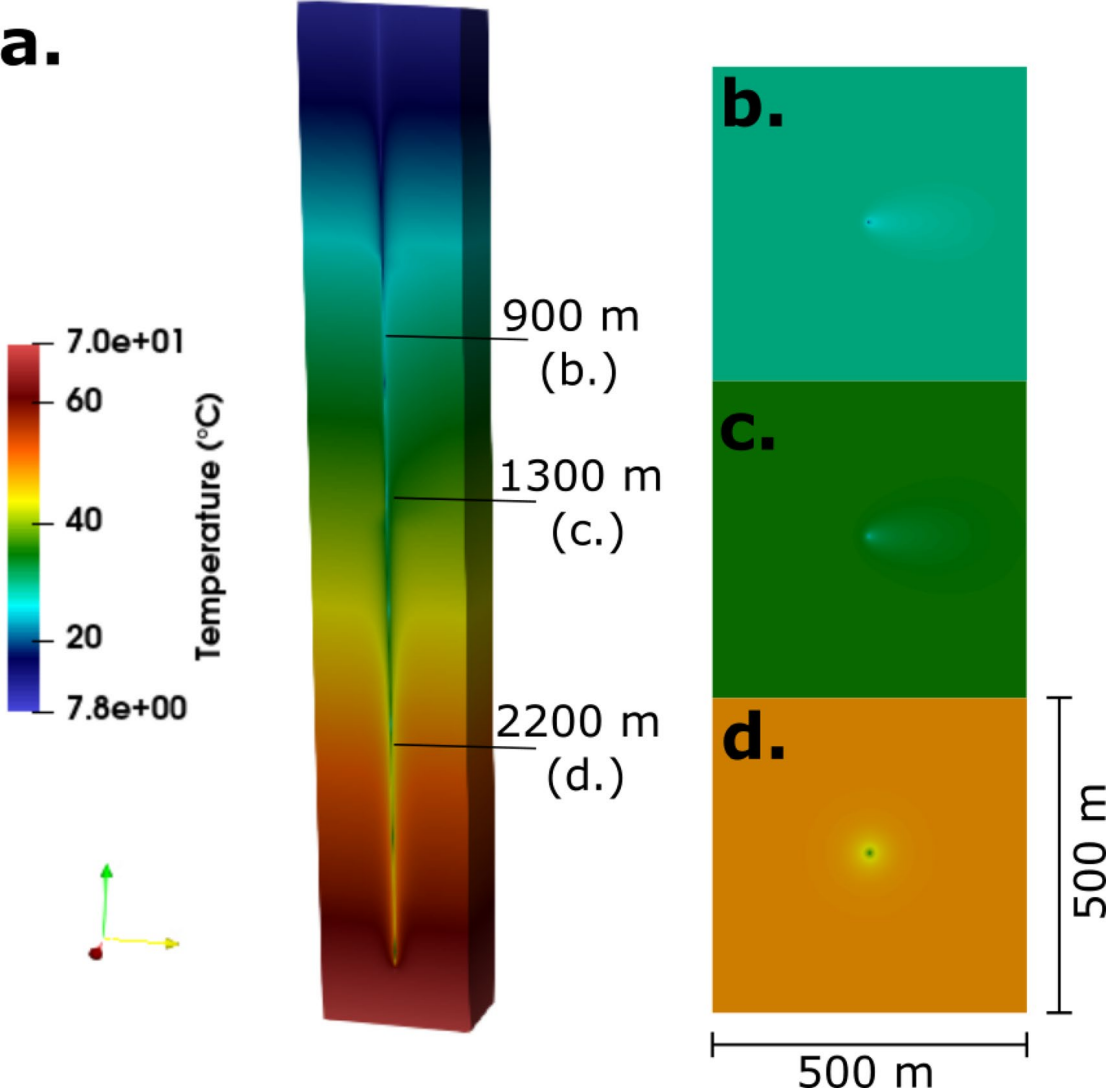
(**a**) 3D advective scenario with plan view plots at (**b**) 900 m through the Helsby Sandstone Formation, (**c**) 1300 m through the Wilmslow Sandstone Formation (un-silicified) and (**d**) 2200 m through the Manchester Marls Formation.

As highlighted in Fig. 7, there is a minor impact on the thermal performance in contrast to the conductive only scenario. A temperature and thermal power increase of 0.24 °C and 4.96 kW was observed, respectively. This occurs as the cold plume is advected away from the DBHE in aquifer layers with higher Darcy velocities. This can be observed in Fig. 8 where the thermal plume propagation reaches proximal to the boundary of the domain (c. 210 m) for both the Helsby (Fig. 8b) and Wilmslow Sandstone Formations (Fig. 8c), whilst for the Manchester Marls Formation (Fig. 8d) the Darcy velocity is low and thermal propagation is near symmetrical around the DBHE.

##### Establishing optimum engineering conditions

**Fig. 9:**
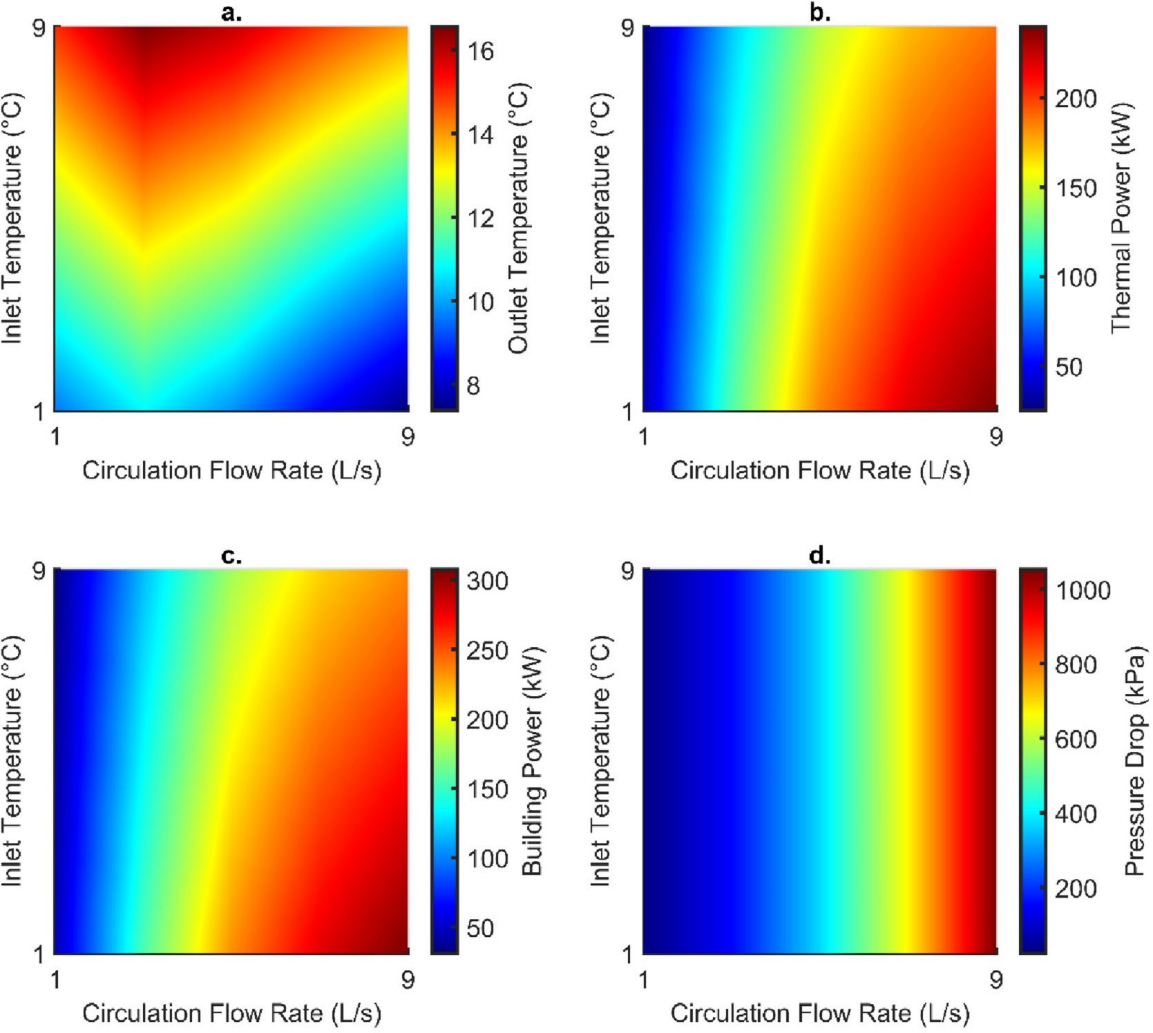
(**a**) Outlet temperature, (**b**) thermal power, (**c**) building power and (**d**) pressure drop recorded at the end of the simulation for varying engineering parameters. Recorded at the end of the simulation.

**Fig. 10:**
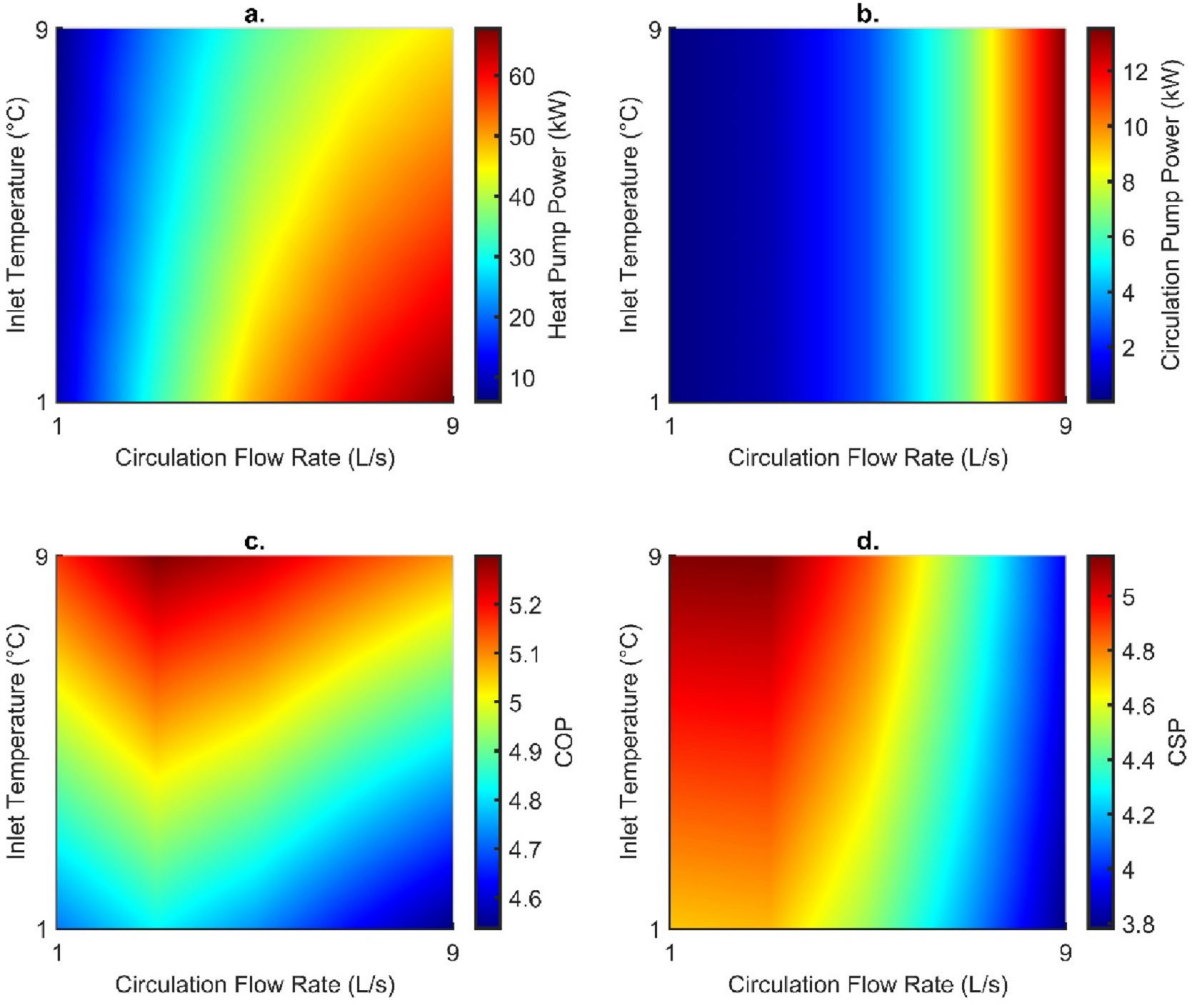
(**a**) Heat pump power, (**b**) circulation pump power, (**c**) coefficient of performance (COP) and (**d**) coefficient of system performance (CSP) recorded at the end of the simulation for varying engineering parameters.

**Fig. 11:**
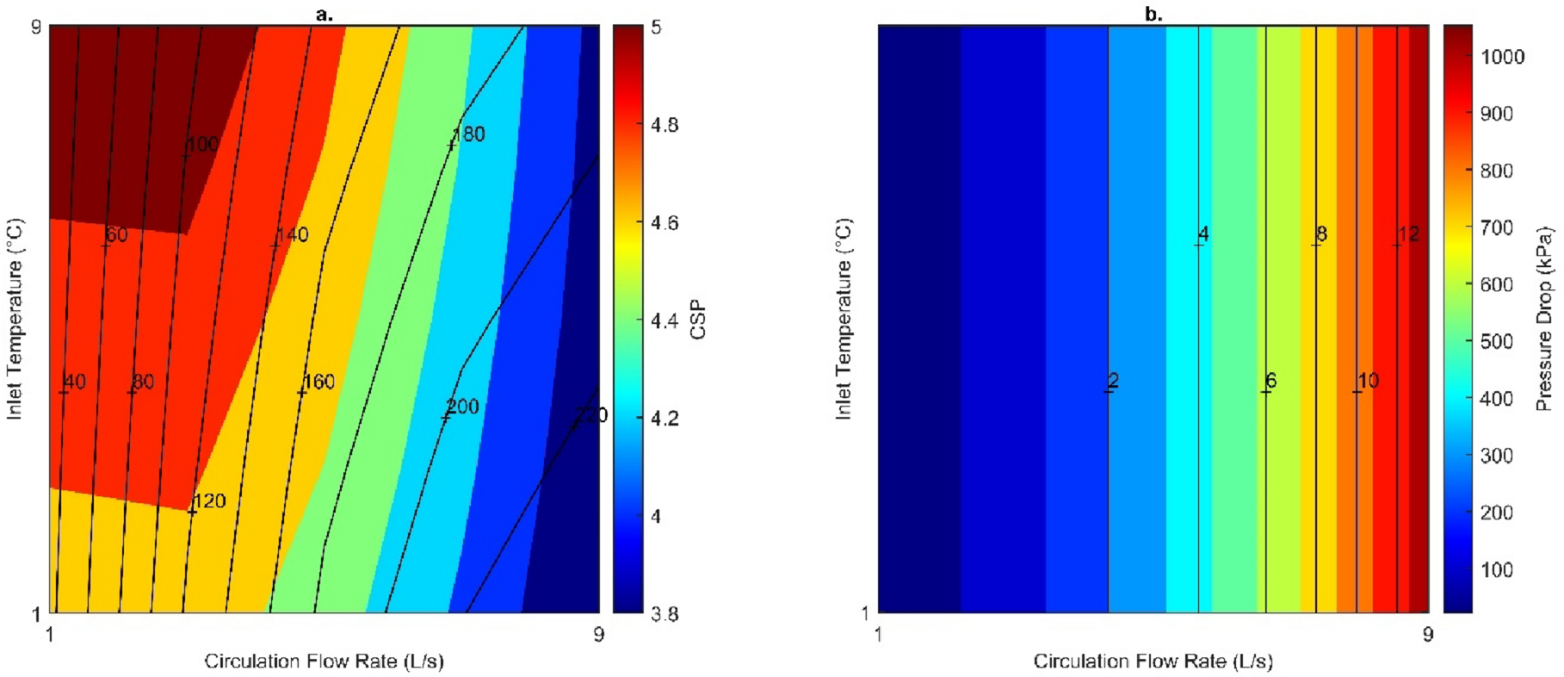
(**a**) Coefficient of system performance plotted with an overlay of thermal power (kW) and (**b**) pressure ddrop with an overlay of circulation pump power (kW) for varying flow rates and inlet temperatures recorded at the end of the simulation.

Mass flow rate and inlet temperature were varied to establish the most suitable conditions to operate the DBHE. Other engineering parameters can influence the DBHE performance, but due to the recompletion of existing infrastructure it would not be possible to alter parameter such as the geometry/configuration of the casing and thermal properties of the DBHE materials. The key influence of engineering parameters is highlighted in Figs. 9 and 10; although it should be noted that they be regarded as the minimum outputs as they are based on temperature data recorded at the end of the simulation. Highest outlet temperature corresponds to a low flow rate of 3 L/s; but both the thermal power and building power increase with lower inlet temperatures and higher flow rates. Due to the simplified method of calculating pressure drop, this increased under the influence of flow rate only. The greatest thermal power from the DBHE was obtained as 189 kW for an inlet temperature of 1 °C and flow rate of 9 L/s. This is also the case for the building power which was recorded as 307 kW after fluid passes through a heat pump. When investigating the parasitic losses of the heat pump for upscaling thermal energy to more useable outlet temperatures it follows a similar trend that colder outlet temperatures from the DBHE (from lower inlet temperatures and faster flow rates) result in greater parasitic losses (Fig. 11).

Other parasitic losses in the system occur from pressure drop and through the increased power required to operate the circulation pump. These two parameters are linked directly linearly, with increasing pressure drop resulting in greater power consumption. Therefore, the higher flow rates result in greater pressure drop (1.05 MPa for 9 L/s) and electricity consumption (13.5 kW). To achieve a high system efficiency (COP or CSP) the corresponding parasitic losses should be minimised through greater inlet temperatures and lower circulation flow rates; however, this can minimise the maximum thermal power the system can operate at. The optimal CSP is thus 5.15 for the flow rate of 3 L/s with an inlet temperature of 9 °C, this is because there is reduced thermal short circuiting at 3 L/s in contrast to 1 L/s and higher outlet temperatures are recorded.

## Discussions

Palaeoclimatic corrections to heat flow must be factored into any studies determining the geothermal potential of prospective areas. Neglecting these can significantly impact the estimation of temperature with depth through static modelling studies, or through transient numerical modelling. This study highlighted that the uncorrected heat flow was 12% lower than the corrected, leading to substantial drops in the modelled steady state temperature gradient with depth. Furthermore, this could lead to a reduction in the modelled thermal power by > 10% which is critical to gauge when determining the economic viability when repurposing a borehole. This can have wider implications on the system efficiency and the ratio of thermal power to parasitic losses from the borehole. Higher thermal powers from the borehole will allow higher coefficients of system performance, and lower ratios of parasitic losses to thermal power obtained from the subsurface. Furthermore, the magnitude of palaeoclimatic corrections across the UK can be far greater up to ~ 30 mWm^− 2 ^^33^. This is much larger than this study highlighting that the potential impact on geothermal systems modelled thermal power could well exceed 10%. It is difficult to extrapolate and quantify the exact amount as it will also depend on borehole depth and local subsurface conditions. Future work should aim to investigate the national impacts of heat flow on geothermal systems.

In addition, systems can be optimised using the operational parameters of the flow rate and inlet temperature. Lower flow rates and higher inlet temperatures will maximise the coefficient of system performance as they reduce the parasitic losses in the circulation and heat pump. However, this will lead to increased thermal short-circuiting which will reduce the thermal power produced from the DBHE. To maximise this the flow rate should be increased and the inlet temperature reduced; however, this will lead to greater parasitic losses.

Whilst this study has highlighted that palaeoclimate corrections to heat flow can help to provide more accurate predictions of subsurface temperatures at depth, there is much uncertainty over the quality of data that exists in the subsurface. This includes, but is not limited to, lack of thermo-physical parameters modelled in-situ, and ambiguity in corrections of temperature measurements with depth (i.e., using a Horner correction)^90^. Thus, the thermo-physical and hydrogeological data in this study was based off regional to national UK values for lithology from literature. If bottom hole temperatures were uncorrected, this could also contribute to the underestimation of heat flow across the basin. Recently a new data set has also been released, with it highlighting a different bottom hole temperature after a period of re-equilibrium of 63.4 °C^91^ which is a very close fit to the estimated bottom hole steady-state temperature in this study of 64.28 °C. The root mean squared error also reduces to 1 °C for the corrected model assuming the new bottom hole temperature. There are also several other factors that can cause near-surface changes in temperature – such as topographic effects, convection, erosion, urban heat island effects and heat refraction^42,93,94^.

Geothermal data measured in the Cheshire Basin should be scrutinised and necessary corrections applied to account for perturbations in the surface thermal state, or discrepancies within the measured data, that have previously been unaccounted for. There is a scarcity in reliable, robust data measured from boreholes in the Cheshire Basin. Undertaking approaches such as that in the present study are necessary to better-understand the magnitude of the deep geothermal resource in the Cheshire Basin.

The stratigraphy encountered by boreholes in the Cheshire Basin, within which temperature, thermal conductivity, and heat flow have been determined is quite variable. A number of boreholes encountered the Sherwood Sandstone Group, others the Mercia Mudstone. These units have significantly different lithological composition (e.g., sandstone compared to mudstone, marl, and on occasion salt/halite) and therefore different thermal properties. This may be reflected in the spread of geothermal gradient and heat flow values determined for boreholes in the Cheshire Basin.

There are also connotations associated to the impact of regional groundwater flow. Groundwater flow (i.e., forced convection) was shown to have a minor influence on the delivery of thermal power from DBHEs over varying lithological layers and Darcy velocities. There was a slight increase in outlet temperature and, thus, thermal power of 5 kW. This agrees with past studies which draw similar conclusions^13,21^. While the increase in performance was minor, there could be greater connotations if developed as a DBHE array. The cooling of the subsurface and increased advective transport would lead to thermal losses in downstream DBHEs and poorer performance. It would be suggested that any boreholes would be placed in a linear array perpendicular in orientation to the groundwater flow, ensuring no thermal interference or cooling effect. If this were not possible and DBHEs were downstream then any benefits of groundwater flow advecting heat could be lost as the thermal plume would likely reduce performance of downstream boreholes if constructed in proximity to the Knutsford borehole. Thus, spacing should be maximised if this were the case to minimise impact of cooling within the zone of influence which reached 210 m. However, the presence, direction and velocity of groundwater flow at depth is not unequivocal in the basin. Past work has speculated Darcy Velocity could be lower than modelled and it would be controlled in the location by density driven flows associated to the mixing of brines and freshwater^39^. This could strongly impact the performance of the system, and thus, there is further potential for free convection to impact system performance, which has been shown to have similar impact to forced convection^92^.

## Conclusions

This study has investigated the potential influence of using palaeoclimate corrections to determine a corrected heat flow prior to modelling of transient DBHEs. The Knutsford-1 Borehole in the Cheshire Basin was used as a case study as there is potential to re-enter this borehole and repurpose it as a DBHE at low-cost. Subsequently, a detailed lithologically layered model was developed simulating both conductive and advective heat transfer in the surrounding formations around the DBHE, before aiming to optimise engineering parameters. The following key conclusions can be drawn:

1. Heat flow in the Cheshire Basin appears to be widely underpredicted. In the case of the Knutsford-1 borehole the uncorrected heat flow was calculated as 46 mW/m^2^, which is lower than the corrected value of 52 mW/m^2^. This impacts the energy available during modelling DBHEs and could have further connotations for predicting the basin-wide geothermal potential.
2. The steady-state setup highlighted that corrected heat flows provide a better fit to measured temperature in the borehole (typically within 1.8 °C). However, there are limitations when analysing results as it appears the borehole was not originally corrected for temperature data (i.e., using a Horner plot to account for cooling from drilling fluid).
3. Transient models indicate that using a corrected or uncorrected heat flow as a basal condition can lead to a decrease in performance by 17 kW. The thermal power recorded at the end of the simulation for the corrected and uncorrected heat flow was 165 and 148 kW, respectively. This is a minimum of a 10% underestimation of the thermal output.
4. Groundwater flow in subsurface aquifers has a minor impact on DBHE performance, this was recorded as a slight increase by 5 kW.
5. Optimum engineering parameters can be determined based upon the system efficiency (COP/CSP) and achievable thermal power (from the DBHE and supplied to the building). The highest thermal power available from the DBHE was 189 kW for an inlet temperature of 1 °C and flow rate of 9 L/s. In contrast, if maximising the system efficiency the highest CSP was 5.15 for the flow rate of 3 L/s with an inlet temperature of 9 °C.

This work fundamentally challenges existing assessments of the UK’s geothermal potential by demonstrating how palaeoclimate corrections significantly alter heat flow estimates. By re-evaluating the Knutsford-1 borehole, the research reveals that heat flows have been systematically underestimated, leading to a misjudgement of geothermal viability. Correcting for past climatic influences translates into a substantial 10% boost in thermal power output for deep borehole heat exchangers – critical for advancing sustainable energy solutions. These findings not only strengthen the economic case for repurposing legacy boreholes but also underscore the urgent need to integrate palaeoclimate adjustments into geothermal feasibility studies worldwide.

## Data Availability

Data can be made available upon reasonable request to the corresponding author.

## List of symbols

$\:{T}_{r}$: Temperature of the rock
$$\:t$$: Time
$$\:\varnothing\:$$: Porosity
$$\:\rho\:$$: Density
$\:c$: Specific heat capacity
$\:\text{v}$: Velocity of groundwater/fluid
$\:{H}_{s}$: Source or sink
$\:{\varPhi\:}_{gs}$: Thermal resistance between rock and cementitious grout
$$\:Q$$: Flow rate
$$\:Re$$: Reynolds number
$\:COP$: Coefficient of performance
$\:CSP$: Coefficient of system performance
$$\:q$$: Heat flow

## Subscripts

r: Solid rock
$$\:g$$: grout/cement
$$\:in$$/i: inlet
$$\:out/o$$: outlet
